# Earth Observation, Spatial Data Quality, and Neglected Tropical Diseases

**DOI:** 10.1371/journal.pntd.0004164

**Published:** 2015-12-17

**Authors:** Nicholas A. S. Hamm, Ricardo J. Soares Magalhães, Archie C. A. Clements

**Affiliations:** 1 Faculty of Geo-Information Science and Earth Observation (ITC), University of Twente, Enschede, The Netherlands; 2 School of Veterinary Science, University of Queensland, Brisbane, Australia; 3 Child Health Research Centre, University of Queensland, Brisbane, Australia; 4 Research School of Population Health, The Australian National University, Canberra, Australia; National Institute of Parasitic Diseases, CHINA

## Abstract

Earth observation (EO) is the use of remote sensing and in situ observations to gather data on the environment. It finds increasing application in the study of environmentally modulated neglected tropical diseases (NTDs). Obtaining and assuring the quality of the relevant spatially and temporally indexed EO data remain challenges. Our objective was to review the Earth observation products currently used in studies of NTD epidemiology and to discuss fundamental issues relating to spatial data quality (SDQ), which limit the utilization of EO and pose challenges for its more effective use. We searched Web of Science and PubMed for studies related to EO and echinococossis, leptospirosis, schistosomiasis, and soil-transmitted helminth infections. Relevant literature was also identified from the bibliographies of those papers. We found that extensive use is made of EO products in the study of NTD epidemiology; however, the quality of these products is usually given little explicit attention. We review key issues in SDQ concerning spatial and temporal scale, uncertainty, and the documentation and use of quality information. We give examples of how these issues may interact with uncertainty in NTD data to affect the output of an epidemiological analysis. We conclude that researchers should give careful attention to SDQ when designing NTD spatial-epidemiological studies. This should be used to inform uncertainty analysis in the epidemiological study. SDQ should be documented and made available to other researchers.

## Introduction

Earth observation (EO) of the environment has found increasing application in epidemiology and public health over the past 40 years [1–3]. It has been used mainly to provide data on the biological and physical environmental variables that determine the distribution of infectious disease, either directly or through their influence on the host, vector, or pathogen habitat. The use of EO in the study of neglected tropical diseases (NTD) is receiving increased attention [3–8].

A characteristic of the life stages of NTDs such as leptospirosis, echinococcosis, schistosomiasis, soil-transmitted helminth (STH) infections, lymphatic filariasis, and onchocerciasis is their strong link to the physical environment, in that environmental factors contribute to the population dynamics of the parasite life stages, intermediate hosts, and vectors [9–15]. For example, it has long been known that the development and survival of *Ascaris lumbricoides* and *Trichuris trichiura* is maximised at a temperature of 28°C to 32°C and that of hookworm at a temperature of 20°C to 30°C [16]. Accordingly, environmental variables are used as inputs into spatial-epidemiological analyses of NTDs. Beck et al. [9] listed 19 variables of interest relating to land cover and land use (also land cover and land use change), vegetation type and phenology, water (including permanent and ephemeral water bodies, flooding, inundated vegetation, soil moisture, and wetlands), and meteorology (precipitation, vapour pressure deficit, and temperature). Other variables of interest include elevation and soil type. Similar variables have been proposed by other authors [3,5,7,8,13,15,17–19].

Spatial-epidemiological analyses of NTD distributions proceed by estimating empirical relationships between epidemiological indicators of disease occurrence (e.g., prevalence and intensity of infection) and environmental and/or socioeconomic variables that are usually modelled as covariates. The purpose of such models is either to provide insight into the factors that influence the spatial distribution of disease or to use the observed empirical relationships between disease and the environment for spatial prediction. Maps based on spatial predictions can serve an important practical purpose, because they can be used to target interventions (e.g., drug treatments) geographically [20].

Recently, broader objectives have emerged for EO applications in NTD epidemiology. A wider range of diseases require attention [21]; there is also an increasing focus on multiple disease outcomes and, in the case of parasitic NTDs, infection intensity and coinfection [22–24] and their associated morbidity [25–27]. These may require different environmental covariates at different spatial and temporal scales. There is an interest in using spatial-epidemiological approaches in an operational context to facilitate efficient surveillance [20,28] and to monitor and evaluate intervention measures [29]. Furthermore, the spatial distribution of disease pathogens, vectors, and hosts are known to change in relation to land cover and land use change [13,30] and are expected to change further in response to climate change [31]. Obtaining and assuring the quality of the relevant spatially and temporally indexed environmental, socioeconomic, and health data, and developing the tools to analyse them, remain important challenges [28]. Finally, it is necessary to evaluate competing modelling approaches and to assess the value of EO in infectious disease studies [32].

During the 21st century, the volume and diversity of remotely sensed and in situ environmental data have increased enormously [33]; however, there have been criticisms that the choice of dataset is often guided by factors such as ease of use, availability, and price, rather than scientific suitability [1,32,34]. The objective of this paper is to review briefly the EO products currently used in studies of NTD epidemiology and to discuss fundamental issues relating to spatial data quality (SDQ), which limit the utilization of EO and pose challenges for its more effective use. This differentiates this review from previous reviews on EO for infectious disease applications. SDQ is important both for the selection of suitable datasets for a NTD study and for evaluating uncertainty in the results of that study.

To inform this review, we undertook a structured literature search focusing on four NTDs: leptospirosis, echinococcosis, schistosomiasis, and STH infections. These are important NTDs that are associated with different environmental determinants and different transmission pathways. We have focused mainly on these four NTDs, although we have drawn on studies of other diseases where they inform our discussion. The strategy for the literature search is explained in Box 1.

**Box 1. Strategy for Literature Search**

We conducted our literature search using Web of Science (Core Collection + Medline) and augmented this with searches of PubMed and PubMed Central. We focused on journal articles rather than conference proceedings. Only articles published in English were included. The date range was 1 January 1980 to 30 May 2015. The primary search was conducted by combining the technical terms related to Earth observation with the four chosen NTDs, e.g., (“remote sensing” AND schistosomiasis). The full list of terms is given in Table 1. This gave the primary list of articles for this review.

We also conducted a secondary search using the environmental terms. This was necessary because several authors do not mention the EO keywords in the abstract or keywords, even if they used these technologies in their research. The secondary search yielded a much longer list of articles, many of which were not relevant. We scanned the abstracts of these articles and then reviewed the most relevant articles. Additionally, we discovered additional references within the articles that we read, as well as through our wider experience. Finally, we searched for articles that addressed the term “spatial data quality” in the context of the four NTDs.

Our search yielded 24 articles for echinococcosis, 15 articles for leptospirosis, 32 articles for soil-transmitted helminths, and 88 articles for schistosomiasis. The search on spatial data quality did not reveal any articles, although we did find one article focusing on malaria and anaemia [35]. These articles were used to inform the review, although we have incorporated wider literature where it is appropriate to do so. In particular, when describing the relevant EO datasets or explaining the issues in spatial data quality, we have gone to the original, most relevant references.

**Table 1:** Search terms used in the literature review. Each disease (each row in column 1) was combined with each group of technical or environmental terms (rows in columns 2 and 3). For details see Box 1.

Disease	Technical	Environmental
echinococcosis, *Echinococcus*	remote sensing, remotely sensed, earth observation, satellite imagery	land cover, land use
schistosomiasis, *Schistosoma*	Landsat, AVHRR, MODIS, ASTER, MERIS, SEVIRI, EUMETSAT, ENVISAT, IRS, TERRA, AQUA, CBERS	Elevation
STH, soil-transmitted helminths, soil-transmitted helminthiasis, hookworm, ascariasis, *Ascaris*, trichuriasis, *Trichuris*	NDVI, normalized difference vegetation index, normalised difference vegetation index	temperature, weather, climate, precipitation, rainfall
leptospirosis	land surface temperature, LST	leaf area index, vegetation, biomass, vegetation biomass

## Earth Observation

The term Earth observation (EO) has commonly been used interchangeably with remote sensing (RS); however, current use of the term is broader and includes in situ observations of the environment [36,37]. EO products may be compiled from RS, in situ data, or some combination of the two. In their conceptualization of Observations and Measurements, the Open Geospatial Consortium (OGC) takes a broader view. They define an observation as “an act associated with a discrete time instant or period through which a number, term or other symbol is assigned to a phenomenon. It involves application of a specified procedure, such as a sensor, instrument, algorithm or process chain” [38]. As such, an observation could be a direct measurement (e.g., thermometer reading), a remotely sensed measurement, or the output of a process chain. The process chain could be routine processing from digital numbers to give a product such as the normalized difference vegetation index (NDVI) or the output from a complex environmental process-based simulator (e.g., weather prediction). This conceptualization is useful because it provides a common platform for conceptualizing data produced using different processes. Note that a disease map, a common output of a spatial epidemiological investigation, is itself an observation (although not an EO). Disease maps can, and have, been used as an input to a subsequent analysis [26]. In this paper, we adopt the above broad interpretation of EO as providing data that relate to the environment. We focus mainly on RS and products derived from RS data, although datasets derived from in situ observations are also considered.

Clear overviews of RS for the epidemiologist are provided by Curran et al. [39] and Hay [40]. Of particular interest is the spatial resolution (pixel size) and the repetivity (the time interval after which a given area is revisited, also called revisit time). We classify spatial resolution as very fine (VFR) (<10 m pixel size), fine (10 to 100 m), moderate (100 to 1,000 m), and coarse (1,000 to 10,000 m). Coarser-resolution sensors generally have shorter repetivities, whereas finer-resolution sensors have longer repetivities or acquire data on demand. Data from very fine-resolution sensors are generally only available at a cost, whereas data from several fine- to coarse-resolution sensors are available freely. A list of sensors commonly used in epidemiology can be found in Table 3 of Kalluri et al. [3] and is augmented by Table 2 (this paper), which includes derived EO products.

**Table 2:** Remotely sensed data and derived products commonly used in epidemiology. These are all global products, and researchers can obtain subsets for their study area. See S1 Text for details.

Sensor	Data product	Variable	Pixel size/resolution (square)	Temporal resolution
AVHRR	GIMMS NDVI3g	NDVI	8 km	10-day (since 1981)
	Pathfinder	NDVI	8 km	10-day (since 1981)
	Pathfinder	MIR	8 km	10-day (since 1981)
	Pathfinder	LST	8 km	10-day (since 1981)
	TFA (Pathfinder)	NDVI	8 km	1981 to 2001
	TFA (Pathfinder)	MIR	8 km	1981 to 2001
	TFA (Pathfinder)	LST	8 km	1981 to 2001
MODIS	Vegetation index	NDVI, EVI	250 m to 1 km	16-day
	LST & emissivity	LST	1 km	Daily/8-day
SPOT VGT	Vegetation index	NDVI	1 km	10-day (since 1998)
AVHRR	IGBP DISCover	Land cover	1 km	1992 to 1993
AVHRR	UMd	Land cover	1 km	1992 to 1993
SPOT VGT	GLC2000	Land cover	1 km	2000
MERIS	GlobCover	Land cover	300 m	12/2004 to 6/2006
	GlobCover 2009	Land cover	300 m	2009
MODIS	Landcover	Land cover	500 m	Annual
WFI CBERS-1/2/2B	-	Red, NIR	260 m	5 days, 10/1999 to 6/2010
IRMSS CBERS-1/2/2B	-	Pan, SWIR	80 m	26 days, 10/1999 to 6/2010
	-	TIR	160 m	26 days, 10/1999 to 6/2010
CCD CBERS-1/2/2B	-	Pan, VNIR	20 m	26 days, 10/1999 to 6/2010
HRC CBERS-2B	-	Pan	2.7 m	130 days, 9/2007 to 6/2010
WIFICAM CBERS-4	-	VNIR	64 m	5 days since 12/2014
IRSCAM CBERS-4	-	Pan, NIR, SWIR	40 m	26 days since 12/2014
	-	TIR	80 m	26 days since 12/2014
MUXCam CBERS-4	-	NIR	20 m	26 days, 10/1999 to 6/2010
PANMUX CBERS-4	-	Pan	5 m	52 days since 12/2014
	-	VNIR	10 m	52 days since 12/2014
Landsat 8 OLI	-	VNIR, SWIR	30 m	16-day repetivity (since 2013)
	-	Panchromatic	15 m	16-day repetivity (since 2013)
Landsat 8 TIS	-	TIR	100 m	16-day repetivity (since 2013)
Landsat	GlobeLand30	Land cover	30 m	2000 and 2010
ASTER	GDEM2	Elevation	1 arc-second (approx. 30 m)	Updated periodically
SRTM SIR-C	SRTM DEM v. 3.0	Elevation	3 arc-second (approx. 90 m)	Flown February 2000

TFA: Temporal Fourier Analysis summary [17]

AVHRR: Advanced Vary High Resolution Radiometer

GIMMS NDVI3g: Global Inventory Modeling and Mapping Studies NDVI v3 [41]

Pathfinder product [42]

SPOT VGT: SPOT Vegetation

MERIS: Medium Resolution Imaging Spectrometer

MODIS: Moderate Resolution Imaging Spectroradiometer

ASTER: Advanced Spaceborne Thermal Emission and Reflection Radiometer

Red/V/SWIR/LWIR: red/visible/short-wave infrared/long-wave infrared portion of the electromagnetic spectrum

IGBP DISCover: International Geosphere Biosphere Program DISCover global land cover map [43]

UMd: University of Maryland global land cover map [44]

GLC2000: Global Landcover Map 2000 [45]

Globcover [46]

CBERS: China—Brazil Earth Resources Satellite program (CBERS) [47–49]

WFI: Wide Field Imager Camera

IRMSS: Infrared Multispectral Scanner Camera

CCD: (high resolution) Charge Couple Device camera (HRCC)

WFICAM: Wide Field Imager Camera (sometimes referred to as WFI or AWFI)

IRSCAM: Infrared Medium Resolution Scanner (sometimes referred to as IRMSS or IRS)

MUXCam: Multispectral Camera

PANMUX: Panchromatic and Multispectral Camera

OLI: Operational Land Imager

TIS: Thermal Infrared Sensor

TIR: Thermal infrared portion of the electromagnetic spectrum

GlobeLand30 [50]

GDEM2: Global Digital Elevation Model v. 2.0 [51]

SRTM: Shuttle Radar Topography Mission v. 3.0 [52]

**Table 3:** Contemporary very fine-resolution sensors. Information was taken from Glackin [62] and Toutin [56] and augmented by information obtained from the relevant websites.

Satellite	Spectral bands	Pixel size (m)	Repetivity (days)	Launched	Swath (km)
Cartosat-1	Panchromatic	2.5	5	2005	30
Cartosat-2	Panchromatic	0.8	4	2007	9.6
LISS-4	VNIR	5.8	24	2003	23.9
EROS-B	Panchromatic	0.7	3 to 4	2006	7
KOMPSAT-3	Panchromatic	0.7	3	2012	16.8
	VNIR	2.8	3	2012	16.8
Quickbird	Panchromatic	0.6	3 to 7	2001	16.5
	VNIR	2.4	3 to 7	2001	16.5
Worldview-1	Panchromatic	0.5	2 to 6	2007	17.6
Worldview-2	Panchromatic	0.5	1 to 2	2009	16.4
	VNIR	1.8	1 to 2	2009	16.4
IKONOS	Panchromatic	0.8	1 to 5	1999	11
	VNIR	4	1 to 5	1999	11
Orbview-3	Panchromatic	1	3	2003	-
	VNIR	4	3	2003	-
GeoEye-1	Panchromatic	0.5	3 to 8	2008	15.2
	VNIR	2	3 to 8	2008	15.2
Pleiades 1A/B constellation	Panchromatic	0.7, 0.5	1	2011/2012	20
	VNIR	2	1	2011/2012	20
SPOT-5 HRG	Panchromatic	5	26	2002	60
	VNIR	10	26	2002	60
SPOT-6/7	Panchromatic	1.5	-	2012/2014	60
	VNIR	8	-	2012/2014	60
RapidEye 5-satellite constellation	VNIR	5 (6.5)	1 to 6	2008	77
FORMOSAT-2	Panchromatic	2	1	2004	24
	VNIR	8	1	2004	24
TerraSAR-X	X-band	1	-	2007	10
	X-band	3	-	2007	30

## Applications of Earth Observation in NTD Epidemiology

We distinguish between static and dynamic environmental variables [3,18]. Static variables include land use and land cover (LULC) and digital elevation models (DEM). Dynamic variables include land surface and vegetation seasonal dynamics as well as seasonal meteorological dynamics. Below, we review EO products that provide these variables.

### Land cover and land use mapping

**Fig 1:**
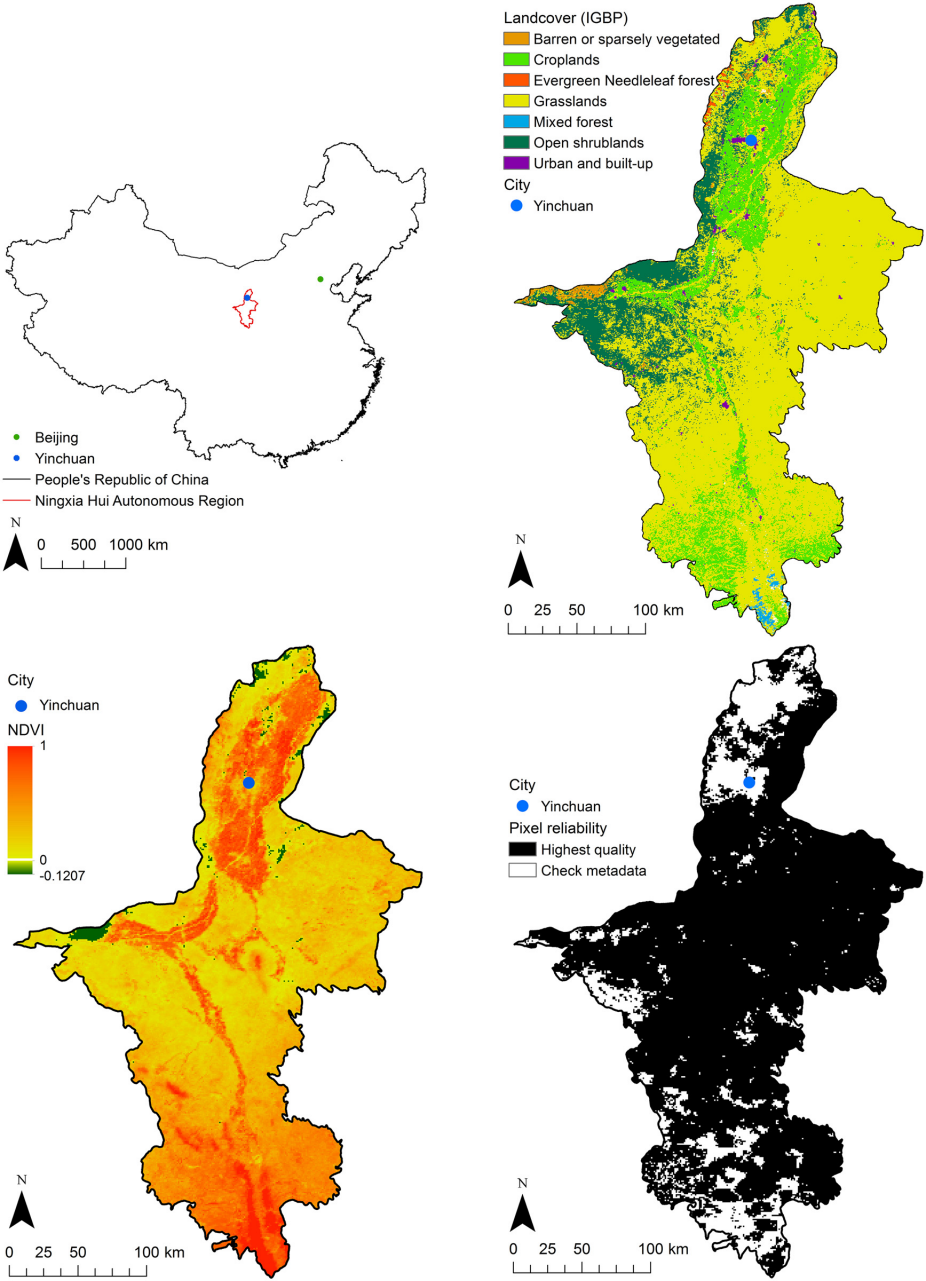
Examples of MODIS products for the Ningxia Hui Autonomous Region (NHAR), China (top left). The top right image shows the 500 m x 500 m annual land cover map (MCD12Q1) for 2012. It uses the IGBP classification scheme (see S1 Text). Only classes covering more than 1% of the NHAR area are shown. The second row shows MODIS (MOD13A3) 1 x 1 km NDVI (bottom left) and pixel reliability (bottom right) maps for July 2012. Pixels flagged as “check metadata” were still of high quality, but flagged because of a moderate atmospheric aerosol load, which can reduce image quality.

LULC includes, for example, vegetation type, human settlements, urban features, and water bodies. Fine-resolution data, provided, for example, by the Landsat series, have been used widely for custom land cover mapping and applied to identification of suitable vector and host breeding sites [2,3,18,53], and have been used for mapping urban areas [54]. There are also several moderate-resolution global land cover maps, which have also seen wide use (e.g., [7,14,55]). An example is shown in Fig 1. Global land cover maps are summarized in Table 2, and an overview is provided in S1 Text.

VFR imagery from aerial surveys has been available for several decades. Over the last 15 years, a variety of VFR satellite imagery (Table 3) has become available [56]. De Castro et al. [57] used aerial photographs to identify potential mosquito breeding grounds in Dar es Salaam, Tanzania. Reis et al. [58] used 16 cm-resolution aerial photography to identify potential leptospirosis risk factors, including open sewers, refuse sites, vegetation, and water bodies in Salvador, Brazil. Limited use has been made of VFR satellite imagery, although Soti et al. [59] used 2.4 m-resolution Quickbird images to detect ponds in a semi-arid area of north Senegal. Addink et al. [60] demonstrated that 2.4 m multispectral Quickbird imagery can be used to delineate the burrows of the great gerbil (*Rhombomys opimus*), an important host for the plague bacterium (*Yersinia pestis*), for a 10 × 6 km test site in Kazakhstan. This study was then extended to a 200 × 250 km area by Wilschut et al. [61] using a Landsat 7 30 m and SPOT-5 2.5 m imagery. An important limitation of VFR data is the lack of a regular acquisition cycle, which limits their utility for monitoring and means that historic data for a given study site may not be available.

### Digital elevation models

DEMs are derived from satellite or airborne RS data [63]. Elevation and the derived variables (such as slope and aspect) may give a measure of habitat suitability or may be correlated with other relevant environmental variables (e.g., temperature, rainfall) [23,64,65]. DEMs can also be used to identify water bodies and potential areas of flooding [58,66]. Freely available DEMs that cover much of the globe at resolutions of 30 m and coarser have been used widely [7,20,24,61,66]. These are listed in Table 2 and summarized in S1 Text. For any given study area, finer-resolution, more accurate DEMs may be available via a private company or government agency [15,58].

### Land surface and vegetation dynamics

The repetivity for fine-resolution sensors is considered too long to monitor environmental dynamics, and their use tends to be restricted to static maps [2,3]. Moderate- and coarse-resolution sensors typically acquire data daily, although they are aggregated over several days for time-series products. Data from these sensors, particularly the NOAA Advanced Very High Resolution Radiometer (AVHRR), have been used for monitoring environmental dynamics [10,67,68]. AVHRR provides a 10-day 8 × 8 km-resolution time series of land surface temperature (LST), middle infrared reflectance (MIR), and NDVI going back to 1981 [41,42]. These variables have been used widely in NTD applications [10,12,20,69]. The time series of monthly NDVI, LST, and MIR data (August 1981 to September 2001) have been processed using temporal Fourier analysis (TFA) and made available to the community by Hay et al. [17]. TFA gives a per-pixel summary of the time series that can be used as a covariate in subsequent analysis [22,69,70]. TFA is of particular interest because it describes the mean, variance, and seasonality in the signal. Other possibilities for summarizing time series include simple summary statistics (e.g., mean, minimum, and maximum) [10,71].

The Moderate Resolution Imaging Spectroradiometer (MODIS) sensor is carried on the NASA Terra and Aqua satellites, launched in 1999 and 2001, respectively [18], as part of the NASA Earth Observing System (EOS). A particular feature of EOS is the provision of a suite of MODIS data products at resolutions of 250, 500, or 1,000 m, with a temporal resolution of 1 day to 1 year. MODIS products are required to be fully documented, including a user guide and quality assurance and validation reports [72–74]. MODIS products are not, however, simply ready to use out of the box. Each product is the outcome of a substantial scientific investigation, and it is necessary to understand the fundamentals of the product and the quality report [75]. MODIS products commonly used in infectious disease studies include land cover type, NDVI, Enhanced Vegetation Index (EVI), and LST (see Table 2), which have seen increased use in recent years [23,55,76]. MODIS 8-day 1 × 1 km time-series for 2001 to 2005 for MIR, NDVI, EVI, and day and night LST have also been processed using TFA and made available to the commnity [75].

LST is a measure of the temperature of the land or vegetation surface. LST is not the same as air temperature, measured using conventional meteorological networks, although it is correlated with it. Temperature is an important control on pathogens, hosts, and vectors. Hence, LST is used widely in NTD studies [3,19,22,77]. NDVI has been very widely used in remote sensing applications over several decades [78] and has been used widely as a covariate for studying the epidemiology of NTDs [3,5,12,34,77]. NDVI allows vegetated and nonvegetated surfaces to be distinguished, and high values are associated with vegetation properties such as biomass, leaf area index (LAI), productivity, and health [78], and is illustrated in Fig 1 where high values are associated with agricultural production. Time series of NDVI values are available from AVHRR (since 1981), MODIS (since 2000), and Satellite Pour l’Observation de la Terre VeGeTation (SPOT VGT) (since 1998), and have been used to study vegetation dynamics and phenology [78]. Furthermore, since healthy vegetation tends to be associated with favourable climatic conditions, it is also used as a surrogate for meteorology [3,67,77]. Despite its succesful application, NDVI is limited because it uses only two wavebands [79], and there are now numerous vegetation and other indices available that use different wavebands and may be more suitable in any given situation [34,80]. Furthermore, there are now MODIS EO products that are based on the modelling of biophysical principles that are generated in a consistent and standardized way [81]. These include vegetation leaf area index (LAI) (MCD15A2 & 3) and net primary productivity (MOD17A3), as well as EVI and land cover dynamics (MCDQ1 & 2). We expect that NDVI will continue to be useful, but to gain a richer understanding of the system under investigation, alternatives should be considered.

### Seasonal meteorological dynamics

Meteorological data are important for NTD studies. Vapour pressure deficit (VPD) can be estimated from AVHRR 8 × 8 km TIR data [82] and MODIS 1 × 1 km LST data [83]. VPD, precipitation, and temperature can also be interpolated from weather station data [82,84]. The Worldclim 1 × 1 km climate summaries [84], which give long-term summaries of monthly precipitation, mean, minimum, and maximum temperature grids for 1950 to 2000, have been used widely in infectious disease studies (with 150+ citations accrued on Web of Science), including NTDs (e.g., [7,14]). In the future, more detailed datasets may become available. For example, Kilibarda et al. [85] published a proof-of-concept global, daily, 1 km-resolution temperature map for 2011 that integrated remotely sensed LST, in situ air temperature, and other remotely sensed covariates.

### Earth observation: New directions

Recent developments in EO may be of future relevance in NTD epidemiology. First, sensors mounted on unmanned aerial vehicles (UAVs/drones) have recently gained increased interest for civilian applications [86]. We found no scientific papers that used UAVs for disease applications, although there is a rapidly developing literature for environmental surveys and urban mapping. Second, Light Detection And Ranging (LiDAR) is used to calculate the distance between the sensor and a target by measuring the response of a reflected laser pulse and can be used to build up highly detailed profiles of surfaces (up to 10 to 20 points per m^2^). Example applications include the development of detailed digital terrain models, 3D vegetation modelling, and the development of 3D models of urban areas [87]. We found very few examples in epidemiology or public health of applications using LiDAR, although Upegui and Viel [88] did use LiDAR for urban mapping in a public health context. Third, in the coming years, the Sentinel missions will be launched by the European Space Agency (ESA). Sentinel-2 (two satellites) will deliver 13 bands in the visible and near infrared (VNIR) and short-wave infrared (SWIR) part of the electromagnetic spectrum. Sentinel 2A was launched on 23 June 2015, and 2B is scheduled for launch in 2016 [89]. Spatial resolution will be 10 to 60 m with a repetivity of 5 days at the equator [90]. Sentinel-3 (three satellites, scheduled for launch in 2015 to 2020 [91]), will carry moderate-resolution sensors with a 1- to 2-day repetivity [92]. Fourth, we expect a wider range of in situ observations (e.g., weather, water level) from official sensor networks and private individuals, to be made available over the internet [93]. The information technology infrastructure to support this “sensor web” is developing rapidly [94]. Fifth, further useful data products may be obtained from integrating multiple remotely sensed and in situ data. An interesting example is provided by Soti et al. [66], who combined fine resolution Quickbird imagery, the Advanced Spaceborne Thermal Emission and Reflection Radiometer Global Digital Elevation Model (ASTER GDEM), and a hydrological model to simulate pond dynamics, which are relevant to mosquito breeding, in north Senegal. Walz et al. [8] call for similar approaches to support schistosomiasis research. Finally, land cover mapping continues to be an active area of research. Attention has turned to the provision of fine resolution global land cover maps [95], such as the 30 m-resolution GlobeLand30 [50,96]. GlobeLand30 was only released to the public in September 2014, and we could not find epidemiological studies that make use of it.

## Important Considerations When Using EO for NTD Studies

Several recent studies of NTD epidemiology have applied Bayesian spatial prediction and emphasize the importance of quantifying uncertainty in the predictions that make up the map [26,70,76]. This prediction uncertainty is based on the Bayesian model and is quantified by, for example, the variance or the width of the credible interval. Prediction uncertainty is location-specific and has implications for the interpretation of the results, for deciding the locations of future surveys, and for intervention planning [20,76,97].

Uncertainty in modelled predictions is affected by uncertainty in both the disease data and the covariates, including the EO data. Considerable attention has been given to uncertainty in the disease data [98–100]. The necessity of addressing uncertainty in the EO predictor variables and propagating it through to epidemiological modelling is noted by Brooker et al. [19] but has not been addressed to date. Below, we focus on issues of uncertainty in EO data in the context of echinococossis, leptospirosis, schistosomiasis, and soil-transmitted helminths. We consider aspects of scale as well as attribute, positional and temporal uncertainty, and their implications for epidemiological studies. We then discuss how these relate to issues of spatial data quality (SDQ). To provide additional support for our discussion, we selected 40 articles (ten for each NTD) and used these as exemplars of whether the four issues of spatial scale, temporal scale, uncertainty, and spatial data quality were addressed properly. These are summarized in the Supporting Information (S2 Text). To avoid bias in our choice of exemplars (i.e., selecting articles that prove a point), we selected the ten articles at random.

### Spatial scale of EO data

EO data are constrained by the measurement process, specifically sampling (support, extent, sample density) and measurement error [101]. Each individual observation occupies a volume or area, referred to as the support (e.g., a 1 × 1 km-resolution MODIS pixel). For raster grid, the support is often referred to as the resolution. The support may also be defined in terms of a buffer drawn around a specific object (e.g., a clinic or other location attached to a disease incidence). A set of observations covers a defined extent (e.g., Queensland, Australia) and is gathered according to a sampling scheme [102]. The property or attribute (e.g., rainfall, NDVI) is subject to measurement error.

EO data may be aggregated or disaggregated to smaller or larger supports [101,103]. Of key importance is that aggregation or disaggregation should be documented explicitly [104] because it leads to new variables with specific statistical properties [105]. Different aggregations (support size and shape) may display different spatial patterns, leading to different conclusions about the variable of interest, a phenomenon known as the modifiable areal unit problem (MAUP) [105–107]. Furthermore, it is common to use multiple EO covariates with different resolutions and where the grids may not be aligned and need to be processed onto the same grid prior to use [55,98]. Such data have been described as incompatible spatial units or spatially misaligned data [108]. We advocate formal, properly documented approaches to aggregation, disaggregation, and spatial misalignments of the type described by Stasch et al. [104] and Atkinson [101], although the tools to implement this need further development.

The scale of variation of disease risk may be fine relative to the often-used moderate- to coarse- resolution EO data [102]. This places a limit on the resolution of the analysis and the resulting disease maps, because if the support is too large, important fine-scale spatial variation may be missed. The appropriate size of support is a function of the objective of study, the research goal, and the analysis method, and may be difficult to identify precisely [109,110]. The support size has received little explicit attention in NTD disease studies, although Soti et al. [59] studied the impact of spatial resolution on the identification of ponds, and Addink et al. [60] explicitly chose 2.4 m-resolution imagery because 0.6 m-resolution imagery was too heterogeneous to permit mapping of the burrows of the great gerbil (*R*. *opimus*), an important reservoir of the Bubonic plague bacterium. Danson et al. [111,112] and Pleydell et al.[6] investigated the impact of buffer size on the relationship between environmental drivers and echinococcus incidence, although they only investigated the buffer size and not the resolution (pixels size) of the associated RS image. Danson et al. [111,112] chose the buffer size that yielded the largest correlation, whereas Pleydell et al. [6] incorporated it as a parameter in their model. Of importance is that the choice of support of the EO data may influence the results. We did not find quantitative methods for choosing the resolution of EO data; however, the researcher needs to consider whether the support of their EO data reflects the variability in the area that they are studying.

Spatial studies in NTD epidemiology cover a range of extents, from a single village [113] or individual suburb (0.5 km^2^) [58] to a small island (140 km^2^) [15], countries [22], and the entire globe [14]. Studies over different extents often come to different conclusions about the environmental and socioeconomic drivers of disease. Simoonga et al. [5] noted that, for schistosomiasis, local studies tend to highlight socioeconomic drivers, whereas larger-extent studies highlight environmental drivers. Similar observations were made by Danson et al. [111] for human alveolar echinococcosis (AE). These conclusions are, however, not generalizable. Reis et al. [58] and Lau et al. [15] were able to identify environmental drivers of leptospirosis, including vegetation, elevation, and distance from refuse sites and sewers. In their study of Chagas disease and schistosomiasis, Kitron et al. [114] showed that disease transmission can be affected by factors outside the extent of the study area. Gracie et al. [115] presented an exploratory study showing that the variability in drivers of leptospirosis was associated with different spatial extents, but did not draw strong conclusions. Clearly, the extent of the study area and the support size should relate to the study objectives and the phenomenon being investigated. In particular, the extent is usually determined by the subject of the investigator’s research (e.g., a suburb in Salvador, Brazil [58]); however, explicit attention is still required here because these choices can affect the results.

The last 15 years have seen the development of sensors with a wide range of spatial resolutions; however, of the 40 papers identified (S2 Text), 27 did not justify the choice of the spatial resolution of the EO data. We recommend that researchers be explicit and consider the implications of these choices. Furthermore, we recommend that the development and application of quantitative methods to identify the relevant extent and support size for a given study objective need further attention.

### Temporal scale of EO data

Spatial sampling considerations of support, extent, and sample density also apply in the temporal domain. Remotely sensed data typically represent a snapshot in time, whereas in situ data may have a defined temporal support (e.g., daily rainfall). Extent refers to the length of the time series. In epidemiological studies, it is common to use temporal aggregates as covariates [28]. For example, the summaries reported in the Worldclim dataset [84] cover 1950 to 2010, giving a temporal support of 50 years. The TFA summaries presented by Hay et al. [17] and Scharlemann et al. [75] cover 20 years (1981 to 2001) and 5 years (2001 to 2005), respectively. In computing and using these summaries, it is assumed the series is stationary (i.e., has a constant mean and variance) over the aggregated support. The consequence of violating this assumption is the estimation of temporal summaries that do not represent the entire aggregated support or the temporal extent of the disease data. This may lead to misleading conclusions about the relationship between disease outcomes and environmental variables. It is, therefore, important that the investigator properly justifies the temporal support of EO data. Considering the 40 identified articles (S2 Text), for 19 articles, there was a mismatch between the timing of the epidemiological and EO data, and only 16 articles explicitly acknowledged the assumption of temporal stationarity. To ensure that this is addressed properly, we recommend that researchers be explicit about the assumptions made and justify whether they are reasonable in the context of their investigation. Possible consequences are outlined below.

The above discussion raises questions for NTD studies. First, we must consider whether the EO data are really stationary over the aggregated support. Notwithstanding potential climate change, land use and land cover can change rapidly, particularly in fast-developing parts of the world [13,30,116]. Second, if the EO data are not stationary, the investigator needs to decide what a suitable temporal support would be. When choosing this, the modifiable temporal unit problem (MTUP) becomes important, particularly when the data show a seasonal periodicity [117]. Hence, both the temporal support and the starting time require careful evaluation, because choices made here may affect the modelled association between the disease data and the EO data. Third, studies tend to use multiple datasets that are defined over temporal supports of different or unspecified lengths and with different start and end dates. In some cases, the temporal dimensions of different EO datasets may not overlap each other or the epidemiological data. The measure of exposure to the environmental conditions may, therefore, be inaccurate, and that this may, in turn, affect the modelled association between the disease data and the EO environmental data. This was noted by Rogers et al. [68], but the effect on the eventual epidemiological analysis remains to be assessed. Finally, we note that modelling disease responses to temporally resolved covariates will require the development and application of spatiotemporal models that can support this [28].

### Uncertainty in EO products

When evaluating spatial data, it is usual to consider the elements of position, time, and attribute. We might measure temperature (the attribute) at a particular location at a particular point in time. Any one of these elements might be uncertain [118]. A set of measurements may be processed further to yield an EO product. For example, the data used to compile the Worldclim EO product are both aggregated temporally and interpolated spatially. Furthermore, EO products based on RS also undergo complex processing, including radiometric and atmospheric correction and geometric correction onto a standard grid [63], as well as further processing that is dependent on the specific product. This will introduce further uncertainty into the final per-pixel attribute value.

Uncertainty may be evaluated by validation against a reference dataset, yielding a measure of accuracy [73,119,120]. Accuracy assessment for land cover mapping based on remote sensing has received extensive attention by Congalton and colleagues [121–123] and by Foody [119]. The reference data should be semantically similar to the data of interest, implying that they should describe the same attribute at the same spatial and temporal support. An extensive system has been developed for the validation of MODIS products [73,74]; for example, the NDVI image shown in Fig 1 has a stated accuracy of ±0.025 [124]. If reference data are not available, other approaches can be taken to evaluate uncertainty. For example, EO products produced using statistical interpolation yield a spatially explicit prediction variance, which is a measure of uncertainty [102,125]. Finally, uncertainty in the input data can be propagated through processing chains to yield a measure of uncertainty in the final result [126,127]. A possible consequence of inaccuracy in EO data is bias in the results of statistical epidemiological analyses. Consider, for example, that the MODIS Collection 5 land cover product (MOD12Q1) (used in, for example, [4,128]) is stated to have an overall accuracy of 75%, and individual classes may be classified less accurately [129].

Ambiguity is an important consideration for the interpretation of land cover maps, because land cover is conceptualized in different ways by different individuals and agencies [130,131]. Fritz and See [132] addressed this when comparing the MODIS land cover products MOD12Q1 and GLC2000 (see Table 2), which use different land cover definitions. They used fuzzy logic and expert opinion to harmonize the class legend of the two maps and to identify areas of uncertainty. Ambiguity is an important issue to consider when making comparisons between studies. We need to be clear whether EO data with the same label really represent the same quantity.

Uncertainty in prediction receives substantial attention in disease mapping studies; however, the uncertainty in the EO data is not usually considered. Of the 40 papers identified (S2 Text), 32 did not consider uncertainty in EO data, and the remaining eight gave only a partial assessment. We recommend further research to identify uncertainty in NTD studies that is associated with uncertainty in EO data, including the choice of EO data products.

### Spatial data quality

The quality of EO data can influence the results of epidemiological analyses. An overview of spatial data quality is provided by Morrison and Veregin [133]. The International Organization for Standardization (ISO) defines data quality elements and procedures for evaluating the quality of geographic data. ISO 19157 [134] defines five quantitative SDQ elements: completeness, logical consistency, positional accuracy, temporal accuracy, and thematic (attribute) accuracy. Completeness refers to omission (missing data) and commission (additional data), and logical consistency refers to the adherence to rules governing the structure of the data [134–136]. Quantitative SDQ elements can be evaluated directly. For example, thematic accuracy can be evaluated against a reference dataset [134]. The quality evaluation may differ between the data producer and the user [135]. The producer evaluates the SDQ elements and determines whether the data meet their specified criteria. The user may have different criteria and may even wish to evaluate the SDQ elements against a different reference dataset.

Quality relates to the “totality of characteristics of a product that bear on its ability to satisfy stated and implied needs” [137]. Hence, to evaluate whether a dataset is fit-for-use, the user (the epidemiologist) needs to evaluate the above data quality elements together with the data specification (including support and extent) and information about the lineage, purpose, and usage. The provision of this information is supported by standards for metadata (ISO standard 19115 [138]) [135,136] as well as its technical implementation (ISO standard 19139 [139]). Lineage, purpose, and usage are often discussed in the context of SDQ [133] and were included as overview elements in earlier ISO standards [137,140], although ISO now consider these part of metadata. These may be used for indirect data quality evaluation based on external knowledge or experience. Historically, metadata standards have been provided by national agencies, although many are now transitioning to the ISO standards. For example, the US Federal Geographic Data Committee (FGDC) now encourages transitioning from the United States Content Standard for Digital Geospatial Information (CSDGM) to ISO 19115 [141].

Standard SDQ metadata have been criticized for being overly complicated, inaccessible, and insufficiently informative to enable a potential user to make a choice about the suitability of a given dataset for their application [37,142,143]. This situation may be exacerbated when the user is not an expert in geoinformation [130,131]. Users tend to use less formal information, such as availability, reputation, cost, and popularity, when making choices about datasets [37,143]. Herbreteau et al. [1] noted the same phenomenon when choices are made about which EO products to include in epidemiological studies, and advocated making choices on more scientific grounds. Tools to help users properly interpret SDQ information include software that allow users to visualize uncertainty and to explore different quality elements [37,144]. Searchable free-text descriptions, including reports from other users, have also been proposed [37,130,143].

Yang et al. [37] proposed that metadata should be organized hierarchically to describe different aspects of the data at different levels of spatial detail. Such an approach is adopted for the MODIS EO products [74], where there is a detailed validation and accuracy assessment that applies to the product as a whole. Furthermore, each individual image has its own specific quality evaluation, as illustrated in Fig 1. Bastin et al. [126] proposed a system that allows documented uncertainty to be propagated through subsequent analysis. Such a system would track processing of the data, including aggregation and disaggregation. Although challenging to implement in the NTD domain, this could bring benefits, including a clear and open documentation of processing steps, which is often lacking in spatial epidemiology papers, and a fuller assessment of uncertainty in epidemiological analyses. Furthermore, we could reason backwards to identify which uncertain EO data and which modelling choices an epidemiological analysis is most sensitive to [126,127]. This could also help to identify the utility of EO products for operational NTD healthcare management [32].

On a more basic level, we recommend that EO datasets and their processing should be clearly described by authors and that a check on this should be part of the peer-review process. Considering the 40 identified articles (S2 Text), the origin of the EO data was not clearly described in 21 articles, and the processing of those data was not clearly described in 22 articles. This journal already requires authors of observational studies in epidemiology to adhere to the STROBE (strengthening the reporting of observational studies in epidemiology [145,146]) statement. A proposal to extend this to include geospatial data was provided by Aimone et al. [35], although that requires further investigation. Finally, we found that the quality of EO data is given little attention in the papers that we reviewed. Of the 40 articles identified (S2 Text), 20 did not discuss the quality of the EO data, and only three papers discussed it thoroughly.

Clements et al. [32] stated that optimal use of EO is restricted by the expertise of the potential user and the difficulty of identifying potentially useful EO data. Restrictions of this nature could be addressed by augmenting widely used datasets, such as those given in Table 2, with user-centred SDQ metadata documenting their suitability for addressing standard questions for specific NTDs. Such an approach would require initial research investment but would benefit operational use in the long term. When an NTD project has specific requirements, an alternative would be to involve geoinformation experts in projects [3,8,34], either as technical consultants or research partners. Finally, there is an increasing demand for VFR RS data [5,32]; however, such data are expensive. We propose that the cost of VFR EO data should be justified in the context of the whole project cost.

### Interaction between uncertainty in EO and NTD data

A full treatment of uncertainty in infection data lies outside the scope of this paper; however, we consider briefly how both the uncertainty in EO and NTD data may interact. We consider two examples concerning scale and positional uncertainty.

Schur et al. [76] and Schur et al. [55] mapped schistosomiasis prevalence in young people at a resolution of 5 × 5 km in west and east Africa, respectively. They then aggregated these maps to estimate endemicity for different administrative units [147]. Aggregation to different administrative units showed different patterns of endemicity and implied different intervention approaches. These studies emphasize three points: first, it is necessary to consider the appropriate spatial resolution for analysis (this was not addressed explicitly); second, there is a MAUP effect, where aggregating to different supports may show different patterns in the data (this was demonstrated by aggregating to different administrative units); finally, the organization of administrative and decision-making units may influence the final map and have consequences for intervention planning. A possible consequence is that localized areas of high endemicity may not be addressed properly.

Cressie and Kornak [148] presented two models of positional uncertainty. Under the coordinate-positioning (CP) model, position is determined in advance but the actual measurement is taken at a different location, for example, due to the use of an imprecise positioning instrument. Under the feature-positioning (FP) model, the attribute is recorded first and a location is assigned later. CP and FP both lead to the response variable being linked to the wrong environmental covariate values [149] but require different solutions [148]. Cressie and Kornak [148] demonstrated a significant effect on geostatistical estimation and prediction and proposed a model to adjust for CP. They did not address FP.

Positional uncertainty has received some attention with respect to species distribution modelling (SDM) in ecology. Here, FP is relevant because animal species are first observed and then later assigned a location. Osborne and Leitao [150] investigated the effect of positional uncertainty in the covariate and the response variable. They introduced a random error into the location of the response variable but a systematic error into the location of the covariate layers. They found that the SDM accuracy was more sensitive to error in the response variable, although they noted that the nature of the errors was quite different. Furthermore, the magnitude of the random error was larger than the systematic error. Naimi et al. [151] concluded that the effect of positonal uncertainty is largest where the range of spatial auto correlation in the covariates is more than three times the standard deviation of the positional uncertainty. Naimi et al. [152] used local indicators of spatial autocorrelation to identify locations where positional uncertainty had a strong effect on species distribution modelling. As with the ecology example, the FP model is relevant in the infectious disease case. This may be a particular problem for historic datasets when precise location data were not gathered and the location was inferred later [65,102]. Additional complications arise because the assigned location (e.g., a home or school) may not be the same as the location where an individual or a group of individuals is exposed to infection [65]. We could not find studies that investigated the effect of positional uncertainty on infectious disease modelling, and we concluded that simulation studies to investigate this effect would be worthwhile.

## Conclusions and Recommendations

EO has found increasing application in public health over the past 40 years and, more recently, in the spatial epidemiology of NTDs. During that time, the research questions have become more complex, and there is an increasing and urgent need to make more informed decisions about the use of suitable EO data in the context of a wider range of health and geospatial tools. At the same time, the volume and diversity of EO data has increased and will continue to increase. In order to make effective use of the data, it is necessary to be critical about what is required and what the relevant spatial and temporal scales are, and to quantify the uncertainty in the EO data as well as the geographically referenced socioeconomic and health data. SDQ should be documented by researchers and made public so that it can be queried to identify suitable datasets, and propagated through epidemiological analyses so that uncertainty in predictions can be evaluated fully. This will require the further development of analytical methods that are appropriate for spatial-temporal data as well as user-friendly software tools. Furthermore, it is necessary to harness recent developments in image analysis and the analysis of time-series data in order to extract useful information from EO data and to model the impact of environmental change on NTDs. Finally, it is necessary to properly evaluate competing modelling approaches and EO data products for both research studies and operational applications.

**Key Learning Points**

- EO has found increasing application to the spatial epidemiology of NTDs. The volume and diversity of EO data has increased and will continue to increase.
- Research questions are becoming increasingly complex, and there is an urgent need to make more informed decisions about the use of suitable EO data in the context of a wider range of health and geospatial tools.
- Spatial data quality should be documented by researchers so that it can be queried to identify suitable datasets and propagated through epidemiological analyses to quantify uncertainty.
- It is necessary to properly evaluate competing EO data products both for research and operational purposes. Spatial and temporal scale and uncertainty are key issues to consider.

**Top Five Papers**

1. Atkinson PM, Graham AJ (2006) Issues of scale and uncertainty in the global remote sensing of disease. Adv Parasitol 62: 79–118. 10.1016/s0065-308x(05)62003-9.
2. Bastin L, Cornford D, Jones R, Heuvelink GBM, Pebesma E, et al. (2013) Managing uncertainty in integrated environmental modelling: The UncertWeb framework. Environ Model Software 39: 116–134. 10.1016/j.envsoft.2012.02.008
3. Clements ACA, Garba A, Sacko M, Toure S, Dembele R, et al. (2008) Mapping the probability of schistosomiasis and associated uncertainty, West Africa. Emerging Infect Dis 14: 1629–1632. 10.3201/eid1410.080366
4. Hay SI, George DB, Moyes CL, Brownstein JS (2013) Big data opportunities for global infectious disease surveillance. PLoS Med 10: e1001413. 1001410.1001371/journal.pmed.1001413
5. Schur N, Vounatsou P, Utzinger J (2012) Determining treatment needs at different spatial scales using geostatistical model-nased risk estimates of schistosomiasis. PLoS Negl Trop Dis 6: e1773. 1710.1371/journal.pntd.0001773

## Supporting Information

S1 Text Global land cover maps and digital elevation models. (DOCX)

S2 Text How well do articles address key issues in scale, uncertainty, and spatial data quality? (DOCX)
