# Supplementary material for: Earth Observation, Spatial Data Quality, and Neglected Tropical Diseases
Source: PLoS Negl Trop Dis. 2015 Dec 17;9(12):e0004164. doi: 10.1371/journal.pntd.0004164 (PMC4683053; doi:10.1371/journal.pntd.0004164)
Supplement: S1 Text — (DOCX) [file pntd.0004164.s001.docx]

# S1 Text: Global land cover maps and digital elevation models

Supporting information for

Hamm NAS, Soares Magalhães RJ, Clements ACA (2015) Earth Observation, Spatial Data Quality and Neglected Tropical Disesases. PLoS Negl Trop Dis. doi: 10.1371/journal.pntd.0004164

Table of Contents

[Supporting Information 1](#_Toc388619086)

[Global land cover maps 2](#_Toc388619087)

[Digital elevation models 2](#_Toc388619088)

[References 4](#_Toc388619089)

# Global land cover maps

There exist various moderate-resolution global land cover maps, such as two 1 km global land cover maps for 1992–1993 [[1](#_ENREF_1)]: the International Geosphere–Biosphere Programme Data and Information System (IGBP-DIS) DISCover [[2-4](#_ENREF_2)] and the University of Maryland (UMd) map [[5](#_ENREF_5)]. Both are based on data from the US National Oceanic and Atmospheric Administration (NOAA) Advanced Very High Resolution Radiometer (AVHRR), but use different classification schemes and algorithms. DISCover uses the IGBP 17-class land classification scheme whereas UMd uses a modifed version of this. GLC2000 (global land cover map 2000) is based on the UN Food and Agricultural Organization (FAO) Land Cover Classification Scheme (LCCS) [[6](#_ENREF_6)]. GLC2000 is also a 1 km resolution map and is based on SPOT VEGETATION data. The LCCS was also used for the GlobCover 2006 and 2009 300 m land cover maps. GlobCover uses data from the Medium Resolution Imaging Spectrometer (MERIS) on board the European Space Agency’s (ESA) Envisat satellite [[7](#_ENREF_7)]. Finally, Collection 5 of the Moderate-resolution Imaging Spectroradiometer (MODIS) delivers a 500 m annual land cover product (MCD12Q1). This uses five different classification schemes, including the IGBP and UMd ones, but not LCCS [[8](#_ENREF_8)].

# Digital elevation models

There is a long history of using photogrammetric methods to compile DEMs using cameras mounted on aircraft [[9](#_ENREF_9)]. DEMs may also be compiled from satellite data. Four currently available products that cover large areas of the globe are: GTOPO30, the ASTER Global DEM (GDEM), the Shuttle Radar Topography Mission (SRTM) DEM [[10](#_ENREF_10)] and the Global Land One-km Base Elevation project (GLOBE). GTOPO30, completed in 1997, was compiled from multiple data sources by the United States Geological Survey (USGS) [[11](#_ENREF_11)] (<http://www.eorc.jaxa.jp/JERS-1/en/GFMP/AM-3/docs/html/gtopo30.htm>). GTOPO30 covers the globe with a grid spacing of 30 arc seconds (approximately 1 km). The SRTM was flown in February 2000 and carried the carried the spaceborne imaging radar (SIR-C) [[12](#_ENREF_12)]. The data have been processed using synthetic appeture radar (SAR) interferommetry to produce a DEM with a 3 arc-second spacing (approximately 90 m) for the global landmass from 56° S to 60° N. Successive versions have sought to remove errors and fill voids. NASA released their most recent version (version 3.0) on 20 November 2013 [[13](#_ENREF_13)], which uses the ASTER GDEM2 to fill voids. The most recent version of GDEM is version 2.0 (GDEM2) [[14](#_ENREF_14)], which uses stereo data from the the nadir and aft-looking infrared cameras collected since 2001. GDEM2 covers the land surface between 83° S to 83° N with a 1 arc-second spacing (approximately 30 m). The verticle accuracy is quoted as being between 10 and 25 m (root mean square error) [[14](#_ENREF_14)]. The validation report [[15](#_ENREF_15)] states that, overall, the accuracy is better than version 1 and comparable to SRTM for the conterminous USA. Various comparisons of GDEM and the SRTM DEM have been published [e.g., [10](#_ENREF_10),[16](#_ENREF_16),[17](#_ENREF_17)], although general guidance does not exist about which is preferred for any given location or application. It has been noted that the GDEM2 tends to be more noisy than SRTM and [Hengl and Reuter [18]](#_ENREF_18) recommend aggregating GDEM2 to 90 m. Note that GDEM2 is available for a wider geographic area and is actually used in SRTM3 to fill voids in rugged terrain. GLOBE [[19](#_ENREF_19),[20](#_ENREF_20)] is the Global Land One-km Base Elevation (GLOBE) project to create a database at 30 arc seconds / 1km by compositing elevation datasets from different sources. Finally, for any given study area the researcher may be able to access a finer resolution DEM, compiled from either airborne or satellite data via a government agency or private company. GTOPO30 [[21](#_ENREF_21),[22](#_ENREF_22)], SRTM [[23](#_ENREF_23),[24](#_ENREF_24)], the ASTER GDEM [[25](#_ENREF_25)] and GLOBE [[26](#_ENREF_26)] have all be used in NTD studies.

# References

1. Hansen MC, Reed B (2000) A comparison of the IGBP DISCover and University of Maryland 1km global land cover products. Int J Remote Sens 21: 1365-1373. doi: 10.1080/014311600210218

2. Loveland TR, Belward AS (1997) The IGBP-DIS global 1 km land cover data set, DISCover: first results. Int J Remote Sens 18: 3291-3295. doi: 10.1080/014311697217099

3. Loveland TR, Reed BC, Brown JF, Ohlen DO, Zhu Z, et al. (2000) Development of a global land cover characteristics database and IGBP DISCover from 1 km AVHRR data. Int J Remote Sens 21: 1303-1330. doi: 10.1080/014311600210191

4. GLCC. Available: <http://edc2.usgs.gov/glcc/globdoc2_0.php> [accessed 31 May 2015]].

5. Hansen MC, Defries RS, Townshend JRG, Sohlberg R (2000) Global land cover classification at 1km spatial resolution using a classification tree approach. Int J Remote Sens 21: 1331-1364. doi: 10.1080/014311600210209

6. Bartholome E, Belward AS (2005) GLC2000: a new approach to global land cover mapping from Earth observation data. Int J Remote Sens 26: 1959-1977. doi: 10.1080/01431160412331291297

7. ESA. 2015. GlobCover. Available: <http://due.esrin.esa.int/page_globcover.php> [accessed 30 July 2015].

8. Friedl MA, Sulla-Menashe D, Tan B, Schneider A, Ramankutty N, et al. (2010) MODIS Collection 5 global land cover: Algorithm refinements and characterization of new datasets. Remote Sens Environ 114: 168-182. doi: 10.1016/j.rse.2009.08.016

9. Lillesand T, Kiefer RW, Chipman J (2008) Remote Sensing and Image Interpretation Wiley Chichester.

10. Mukherjee S, Joshi PK, Mukherjee S, Ghosh A, Garg RD, et al. (2013) Evaluation of vertical accuracy of open source Digital Elevation Model (DEM). Int J Appl Earth Obs Geoinf 21: 205-217. doi: 10.1016/j.jag.2012.09.004

11. GTOPO30. Available: <http://www.eorc.jaxa.jp/JERS-1/en/GFMP/AM-3/docs/html/gtopo30.htm> [accessed 20 September 2015].

12. Farr TG, Rosen PA, Caro E, Crippen R, Duren R, et al. (2007) The Shuttle Radar Topography Mission. Rev Geophys 45: RG2004. doi: 10.1029/2005RG000183

13. SRTM. 2013. NASA Shuttle Radar Topography Mission (SRTM) Version 3.0 (SRTM Plus) Product Release. Available: <https://lpdaac.usgs.gov/about/news_archive/nasa_shuttle_radar_topography_mission_srtm_version_30_srtm_plus_product_release> [accessed 20 September 2015].

14. USGS. 2015. Available: <https://lpdaac.usgs.gov/dataset_discovery/aster/aster_products_table> [accessed 20 September 2015].

15. Meyer D (2011) ASTER Global Digital Elevation Model Version 2 – Summary of Validation Results. ASTER GDEM Validation Team.

16. Li P, Shi C, Li Z, Muller J-P, Drummond J, et al. (2013) Evaluation of ASTER GDEM using GPS benchmarks and SRTM in China. Int J Remote Sens 34: 1744-1771. doi: 10.1080/01431161.2012.726752

17. Zhao SM, Cheng WM, Zhou CH, Chen X, Zhang SF, et al. (2011) Accuracy assessment of the ASTER GDEM and SRTM3 DEM: an example in the Loess Plateau and North China Plain of China. Int J Remote Sens 32: 8081-8093. doi: 10.1080/01431161.2010.532176

18. Hengl T, Reuter H (2011) How accurate and usable is GDEM? A statistical assessment of GDEM using LiDAR data. Geomorphometry 2: 45-48. doi:

19. Hastings DA, Dunbar PK (1999) Global Land One-kilometer Base Elevation (GLOBE) Digital Elevation Model, Documentation, Volume 1.0. 325 Broadway, Boulder, Colorado 80305, U.S.A: National Oceanic and Atmospheric Administration (NOAA), National Geophysical Data Center.

20. GLOBE Task Team and others (Hastings DA, Paula K. Dunbar, Gerald M. Elphingstone, Mark Bootz, Hiroshi Murakami, Hiroshi Maruyama, Hiroshi Masaharu, Peter Holland, John Payne, Nevin A. Bryant, Thomas L. Logan, J.-P. Muller, Gunter Schreier, and John S. MacDonald) (1999) The Global Land One-kilometer Base Elevation (GLOBE) Digital Elevation Model, Version 1.0. 325 Broadway, Boulder, Colorado 80305-3328, U.S.A: National Oceanic and Atmospheric Administration, National Geophysical Data Center.

21. Raso G, Matthys B, N'Goran EK, Tanner M, Vounatsou P, et al. (2005) Spatial risk prediction and mapping of Schistosoma mansoni infections among schoolchildren living in western Cote d'Ivoire. Parasitology 131: 97-108. doi: 10.1017/s0031182005007432

22. Raso G, Vounatsou P, McManus DP, Utzinger J (2007) Bayesian risk maps for *Schistosoma mansoni* and hookworm mono-infections in a setting where both parasites co-exist. Geospatial Health 2: 85-96. doi: 10.4081/gh.2007.257

23. Clements ACA, Kur LW, Gatpan G, Ngondi JM, Emerson PM, et al. (2010) Targeting Trachoma Control through Risk Mapping: The Example of Southern Sudan. PLoS Negl Trop Dis 4: e799. doi: 10.1371/journal.pntd.0000799

24. Wilschut LI, Addink EA, Heesterbeek JAP, Dubyanskiy VM, Davis SA, et al. (2013) Mapping the distribution of the main host for plague in a complex landscape in Kazakhstan: An object-based approach using SPOT-5 XS, Landsat 7 ETM+, SRTM and multiple Random Forests. Int J Appl Earth Obs Geoinf 23: 81-94. doi: 10.1016/j.jag.2012.11.007

25. Soti V, Puech C, Lo Seen D, Bertran A, Vignolles C, et al. (2010) The potential for remote sensing and hydrologic modelling to assess the spatio-temporal dynamics of ponds in the Ferlo Region (Senegal). Hydrol Earth Syst Sci 14: 1449-1464. doi: 10.5194/hess-14-1449-2010

26. Giraudoux P, Raoul F, Pleydell D, Li T, Han X, et al. (2013) Drivers of echinococcus multilocularis transmission in China: small mammal diversity, landscape or climate? PLoS Negl Trop Dis 7: e2045. doi: 10.1371/journal.pntd.0002045
