# Supplementary material for: Earth Observation, Spatial Data Quality, and Neglected Tropical Diseases
Source: PLoS Negl Trop Dis. 2015 Dec 17;9(12):e0004164. doi: 10.1371/journal.pntd.0004164 (PMC4683053; doi:10.1371/journal.pntd.0004164)
Supplement: S2 Text — (DOCX) [file pntd.0004164.s002.docx]

# S2 Text: How well do articles address key issues in scale, uncertainty and spatial data quality?

Supporting information for

Hamm NAS, Soares Magalhães RJ, Clements ACA (2015) Earth Observation, Spatial Data Quality and Neglected Tropical Disesases. PLoS Negl Trop Dis. doi: 10.1371/journal.pntd.0004164

From our structured search we selected 10 papers at random for each disease (40 papers in total). The table (overleaf) shows whether they address the four issues of spatial and temporal scales, uncertainty and spatial data quality. In each column the number of articles and the actual citations are given. Lepto=leptospirosis, Schisto=schistosomiasis, Echino=echinococcosis, STH=soil-transmitted helminths.

|  | Lepto | Schisto | Echino | STH | Total |
| --- | --- | --- | --- | --- | --- |
|  | [1-10] | [11-20] | [21-30] | [31-40] | **40** |
| Spatial scale of EO data |  |  |  |  |  |
| Choice of spatial resolution of EO data not justified | **7**  [1-3,5-7,10] | **9**  [11,13-20] | **5** [22,23,26,27,29] | **6**  [32,34,36-38,40] | **27** |
| Choice of buffer size not justified^[[1]](#footnote-1)^ | **2**  [2,9] | **2**  [11,17] | **1**  [24] | **0** | **5** |
| Choice of spatial extent of study not justified^[[2]](#footnote-2)^ | **0** | **2**  [15,16] | **1**  [26] | **0** | **3** |
| Temporal scale of EO data |  |  |  |  |  |
| Assumption of temporal stationarity not acknowledged. | **1**  [3] | **4**  [11,18-20] | **5**  [23,24,26,27,29] | **6** [31,32,34,35,37,38] | **16** |
| Choice of temporal support not justified | **0** | **3**  [16,19,20] | **4** [23,24,26,27] | **6** [31,32,34,37,38,40] | **13** |
| Mismatch in the timing of acquisition between different EO data | **2**  [1,5] | **4** [14,17,18,20] | **3** [24,26,27] | **3** [31-34] | **12** |
| Mismatch in the timing of acquisition between the EO and epidemiological data | **4** [1,2,5,6] | **5** [14,17-20] | **4** [23,24,26,27] | **6** [31-34,37-39] | **19** |
| Uncertainty in EO products |  |  |  |  |  |
| Uncertainty in EO data not included in the epidemiological modelling | **6** [1-4,7,8] | **8** [11,13-15,17-20] | **9** [22-30] | **9** [31-38,40] | **32** |
| Attribute values not defined clearly. | **2** [1,3] | **5** [11,13,15,19,20] | **2** [23,29] | **7** [32-36,38,39] | **16** |
| Spatial data quality |  |  |  |  |  |
| Origin of EO data not clearly documented (lineage) | **5** [1,3-6] | **5** [11,13-15,20] | **3** [23,27,29] | **8** [31,32,34-36,38-40] | **21** |
| Processing of EO data not clearly documented (lineage) | **6**  [1-6] | **3** [15,19,20] | **6** [22-24,26,29,30] | **7** [32-36,38,39] | **22** |
| Choice of EO data products not clearly justified | **6** [1,2,6-8,10] | **9** [11,13-20] | **6** [22,23,25-27,29] | **6** [32-34,36-38] | **27** |
| No discussion of the quality of EO data | **6** [3,6-10] | **4** [11,15,18,20] | **4** [24,26,27,29] | **6** [32,34,36-38,40] | **20** |
| Partial discussion of the quality of EO data | **3** [1,2,4] | **5** [13,14,16,17,19] | **5** [22,23,25,28,30] | **4** [31,33,35,39] | **17** |
| Extensive discussion of quality of EO data | **1** [5] | **1** [12] | **1** [21] | **0** | **3** |

# References

1. Gracie R, Barcellos C, Magalhaes M, Souza-Santos R, Guimaraes Barrocas PR (2014) Geographical Scale Effects on the Analysis of Leptospirosis Determinants. Int J Env Res Public Health 11: 10366-10383. doi: 10.3390/ijerph111010366

2. Ivanova S, Herbreteau V, Blasdell K, Chaval Y, Buchy P, et al. (2012) Leptospira and Rodents in Cambodia: Environmental Determinants of Infection. Am J Trop Med Hyg 86: 1032-1038. doi: 10.4269/ajtmh.2012.11-0349

3. Lerdthusnee K, Nigro J, Monkanna T, Leepitakrat W, Leepitakrat S, et al. (2008) Surveys of rodent-borne disease in Thailand with a focus on scrub typhus assessment. Integrative Zoology 3: 267-273. doi: 10.1111/j.1749-4877.2008.00100.x

4. Raghavan R, Brenner K, Higgins J, Van der Merwe D, Harkin KR (2011) Evaluations of land cover risk factors for canine leptospirosis: 94 cases (2002-2009). Prev Vet Med 101: 241-249. doi: 10.1016/j.prevetmed.2011.05.010

5. Raghavan RK, Brenner KM, Harrington JA, Jr., Higgins JJ, Harkin KR (2013) Spatial scale effects in environmental risk-factor modelling for diseases. Geospatial Health 7: 169-182. doi:

6. Reis RB, Ribeiro GS, Felzemburgh RDM, Santana FS, Mohr S, et al. (2008) Impact of environment and social gradient on leptospira infection in urban slums. PLoS Negl Trop Dis 2: e228. doi: 10.1371/journal.pntd.0000228

7. Lau CL, Dobson AJ, Smythe LD, Fearnley EJ, Skelly C, et al. (2012) Leptospirosis in American Samoa 2010: Epidemiology, Environmental Drivers, and the Management of Emergence. Am J Trop Med Hyg 86: 309-319. doi: 10.4269/ajtmh.2012.11-0398

8. Socolovschi C, Angelakis E, Renvoise A, Fournier P-E, Marie JL, et al. (2011) Strikes, flooding, rats, and leptospirosis in Marseille, France. Int J Infect Dis 15: E710-E715. doi: 10.1016/j.ijid.2011.05.017

9. Tassinari WS, Pellegrini DC, Sa CB, Reis RB, Ko AI, et al. (2008) Detection and modelling of case clusters for urban leptospirosis. Trop Med Int Health 13: 503-512. doi: 10.1111/j.1365-3156.2008.02028.x

10. Coelho MSZS, Massad E (2012) The impact of climate on Leptospirosis in Sao Paulo, Brazil. Int J Biometeorol 56: 233-241. doi: 10.1007/s00484-011-0419-4

11. Hu Y, Li R, Bergquist R, Lynn H, Gao F, et al. (2015) Spatio-temporal transmission and environmental determinants of Schistosomiasis Japonica in Anhui Province, China. PLoS Negl Trop Dis 9: e0003470. doi: 10.1371/journal.pntd.0003470

12. Tseng K-H, Liang S, Ibaraki M, Lee H, Shum CK (2014) Study of the variation of schistosomiasis risk in Lake Poyang in the People's Republic of China using multiple space-borne sensors for monitoring and modelling. Geospatial Health 8: 353-364. doi:

13. Woodhall DM, Wiegand RE, Wellman M, Matey E, Abudho B, et al. (2013) Use of Geospatial Modeling to Predict *Schistosoma mansoni* Prevalence in Nyanza Province, Kenya. Plos One 8: e71635. doi: 10.1371/journal.pone.0071635

14. Raso G, Matthys B, N'Goran EK, Tanner M, Vounatsou P, et al. (2005) Spatial risk prediction and mapping of *Schistosoma mansoni* infections among schoolchildren living in western Cote d'Ivoire. Parasitology 131: 97-108. doi: 10.1017/s0031182005007432

15. Davis GM, Wu WP, Chen HG, Liu HY, Guo JG, et al. (2002) A baseline study of importance of bovines for human *Schistosoma japonicum* infections around Poyang Lake, China: Villages studied and snail sampling strategy. Am J Trop Med Hyg 66: 359-371. doi:

16. Zhang Z, Bergquist R, Chen D, Yao B, Wang Z, et al. (2013) Identification of parasite-host habitats in Anxiang county, Hunan Province, China based on multi-temporal China-Brazil earth resources satellite (CBERS) images. PloS ONE 8: e69447. doi: 10.1371/journal.pone.0069447

17. Malone JB, Yilma JM, McCarroll JC, Erko B, Mukaratirwa S, et al. (2001) Satellite climatology and the environmental risk of *Schistosoma mansoni* in Ethiopia and east Africa. Acta Trop 79: 59-72. doi: 10.1016/s0001-706x(01)00103-6

18. Kristensen TK, Malone JB, McCarroll JC (2001) Use of satellite remote sensing and geographic information systems to model the distribution and abundance of snail intermediate hosts in Africa: a preliminary model for *Biomphalaria pfeifferi* in Ethiopia. Acta Trop 79: 73-78. doi: 10.1016/s0001-706x(01)00104-8

19. Pullan RL, Bethony JM, Geiger SM, Cundill B, Correa-Oliveira R, et al. (2008) Human Helminth Co-Infection: Analysis of Spatial Patterns and Risk Factors in a Brazilian Community. PLoS Negl Trop Dis 2: e352. doi: 10.1371/journal.pntd.0000352

20. Schrader M, Hauffe T, Zhang Z, Davis GM, Jopp F, et al. (2013) Spatially Explicit Modeling of Schistosomiasis Risk in Eastern China Based on a Synthesis of Epidemiological, Environmental and Intermediate Host Genetic Data. PLoS Negl Trop Dis 7: e2327. doi: 10.1371/journal.pntd.0002327

21. Danson FM, Craig PS, Man W, Shi DH, Giraudoux P (2004) Landscape dynamics and risk modeling of human alveolar echinococcosis. Photogramm Eng Remote Sensing 70: 359-366. doi:

22. Pleydell DRJ, Yang YR, Danson FM, Raoul F, Craig PS, et al. (2008) Landscape composition and spatial prediction of alveolar echinococcosis in southern Ningxia, China. PLoS Negl Trop Dis 2: e287. doi: 10.1371/journal.pntd.0000287

23. Hu HH, Wu WP, Guan YY, Wang LY, Wang Q, et al. (2014) A village-based multidisciplinary study on factors affecting the intensity of cystic echinococcosis in an endemic region of the Tibetan plateau, China. Epidemiol Infect 142: 1214-1220. doi: 10.1017/s0950268813002124

24. Staubach C, Thulke HH, Tackmann K, Hugh-Jones M, Conraths FJ (2001) Geographic information system-aided analysis of factors associated with the spatial distribution of *Echinococcus multilocularis* infections of foxes. Am J Trop Med Hyg 65: 943-948. doi:

25. Burlet P, Deplazes P, Hegglin D (2011) Age, season and spatio-temporal factors affecting the prevalence of *Echinococcus multilocularis* and *Taenia taeniaeformis* in *Arvicola terrestris*. Parasites and Vectors 4: 6. doi: 10.1186/1756-3305-4-6

26. Giraudoux P, Raoul F, Pleydell D, Li T, Han X, et al. (2013) Drivers of *Echinococcus multilocularis* transmission in China: small mammal diversity, landscape or climate? PLoS Negl Trop Dis 7: e2045. doi: 10.1371/journal.pntd.0002045

27. Kantzoura V, Diakou A, Kouam MK, Feidas H, Theodoropoulou H, et al. (2013) Seroprevalence and risk factors associated with zoonotic parasitic infections in small ruminants in the Greek temperate environment. Parasitol Int 62: 554-560. doi: 10.1016/j.parint.2013.08.010

28. Graham AJ, Danson FM, Giraudoux P, Craig PS (2004) Ecological epidemiology: landscape metrics and human alveolar echinococossis. Acta Trop 91: 267-278. doi: 10.1016/j.actatropica.2004.04.005

29. Lagapa JTG, Oku Y, Kaneko M, Ganzorig S, Ono T, et al. (2009) Monitoring of environmental contamination by *Echinococcus multilocularis* in an urban fringe forest park in Hokkaido, Japan. Environ Health Prevent Med 14: 299-303. doi: 10.1007/s12199-009-0083-z

30. Danson FM, Graham AJ, Pleydell DRJ, Campos-Ponce M, Giraudoux P, et al. (2003) Multi-scale spatial analysis of human alveolar echinococcosis risk in China. Parasitology 127: S133-S141. doi: 10.1017/s0031182003003639

31. Brooker S, Hay SI, Tchuente LAT, Ratard R (2002) Using NOAA-AVHRR data to model human helminth distributions in planning disease control in Cameroon, West Africa. Photogramm Eng Remote Sensing 68: 175-179. doi:

32. Chammartin F, Scholte RGC, Malone JB, Bavia ME, Nieto P, et al. (2013) Modelling the geographical distribution of soil-transmitted helminth infections in Bolivia. Parasites & Vectors 6: 152. doi: 10.1186/1756-3305-6-152

33. Pullan RL, Brooker SJ (2012) The global limits and population at risk of soil-transmitted helminth infections in 2010. Parasites & Vectors 5: 81. doi: 10.1186/s13071-015-0732-6

34. Lai YS, Zhou XN, Utzinger J, Vounatsou P (2013) Bayesian geostatistical modelling of soil-transmitted helminth survey data in the People's Republic of China. Parasites & Vectors 6: 359. doi: 10.1186/1756-3305-6-359

35. Brooker S, Kabatereine NB, Tukahebwa EM, Kazibwe F (2004) Spatial analysis of the distribution of intestinal nematode infections in Uganda. Epidemiol Infect 132: 1065-1071. doi: 10.1017/s0950268804003024

36. Mudenda NB, Malone JB, Kearney MT, Mischler PD, Nieto PD, et al. (2012) Modelling the ecological niche of hookworm in Brazil based on climate. Geospatial Health 6: S111-S123. doi: 10.4081/gh.2012.129

37. Soares Magalhães RJ, Biritwum NK, Gyapong JO, Brooker S, Zhang YB, et al. (2011) Mapping Helminth Co-Infection and Co-Intensity: Geostatistical Prediction in Ghana. PLoS Negl Trop Dis 5: e1200. doi: 10.1371/journal.pntd.0001200

38. Scholte RGC, Freitas CC, Dutra LV, Guimaraes RJPS, Drummond SC, et al. (2012) Utilizing environmental, socioeconomic data and GIS techniques to estimate the risk for ascariasis and trichuriasis in Minas Gerais, Brazil. Acta Trop 121: 112-117. doi: 10.1016/j.actatropica.2011.10.011

39. Saathoff E, Olsen A, Kvalsvig JD, Appleton CC, Sharp B, et al. (2005) Ecological covariates of *Ascaris lumbricoides* infection in schoolchildren from rural KwaZulu-Natal, South Africa. Trop Med Int Health 10: 412-422. doi: 10.1111/j.1365-3156.2005.01406.x

40. Forrer A, Vounatsou P, Sayasone S, Vonghachack Y, Bouakhasith D, et al. (2015) Risk Profiling of Hookworm Infection and Intensity in Southern Lao People's Democratic Republic Using Bayesian Models. PLoS Negl Trop Dis 9: e0003486. doi: 10.1371/journal.pntd.0003486

1. Buffer size not relevant in all studies. [↑](#footnote-ref-1)
2. Determined by the study objective in many articles. [↑](#footnote-ref-2)
